# Effect of Neoadjuvant Hormone Therapy on Resection Margin and Survival Prognoses in Locally Advanced Prostate Cancer after Prostatectomy Using Propensity-Score Matching

**DOI:** 10.1155/2018/4307207

**Published:** 2018-12-06

**Authors:** Sung Han Kim, Eun Young Park, Jungnam Joo, Jae Young Joung, Ho Kyung Seo, Jinsoo Chung, Kang Hyun Lee

**Affiliations:** ^1^Department of Urology, Center for Prostate Cancer, Research Institute and Hospital of National Cancer Center, Goyang, Republic of Korea; ^2^Biometrics Research Branch, Division of Cancer Epidemiology and Prevention, Research Institute and Hospital of National Cancer Center, Goyang, Republic of Korea

## Abstract

This study aimed to investigate the effect of neoadjuvant hormone therapy (NHT) on resection margin positivity, biochemical-recurrence- (BCR-) free survival, and overall survival (OS) in 176 patients with locally advanced prostate cancer (LAPC) treated with radical prostatectomy using propensity-score matching, including 79 (44.9%) patients treated with the NHT. Fifty pairs of one-to-one propensity-score matching were matched to investigate the pure effect of NHT on resection margin positivity, BCR, and OS with a statistical significance of* p*<0.050. Before matching, NHT, tumor volume percentage, and extracapsular extension were significant factors for resection margin positivity (*p*≤0.001); however, after matching, NHT became insignificant in the multivariate analysis (*p*=0.084). In the survival analysis, NHT was not associated with BCR or OS before and after matching (BCR: hazard ratio, 1.35 and 0.84, respectively; OS: hazard ratio, 1.05 and 0.77, respectively;* p*≥0.539 for all). Conversely, PSA level (HR, 2.23), extracapsular extension (HR, 2.10), and lymphovascular invasion (HR, 1.85) were significant factors for BCR (*p*≤0.001 for all), but none were significant factors for OS in the propensity-score matching analysis (*p*≥0.948). Therefore, NHT was not a significant factor for resection margin positivity, BCR-free survival, and OS before and after propensity-score matching in patients with LAPC.

## 1. Introduction

Prostate cancer (PC) results from an androgen-dependent tumor in males. The androgen-producing mechanism is a key therapeutic objective in the treatment of PC. In 1964, Scott and colleagues introduced the idea of encouraging recurrence-free survival in men with advanced PC by treating with radical prostatectomy (RP) and androgen deprivation therapy or androgen blocking hormonal therapy (HT). Presently, HT is one of the standard therapeutic options for PC in order to inhibit the growth of PC, especially for recurred PC after prostatectomy or advanced PC [1].

The rationale for using HT in the neoadjuvant setting (NHT) is to reduce the size of the tumor before surgery in an effort to improve surgical treatment for locally advanced PC. In combination with RP, NHT has been shown to result in improvements in both clinical and local pathological outcomes, including achieved pathologic T0 with RP [2]. Improvements include downstaging and organ confinement, with a reduction in positive resection margin (RM) rates and a decrease of lymph node involvement [3, 4]. However, because of existing differences in enrolled patients' baseline characteristics in prior studies [1, 4], there still remains uncertainty as to NHT's direct clinical and pathological impact in locally advanced PC prior to RP, especially in the aspects of overall survival (OS) and disease-free survival [1] that the current EAU guideline 2017 does not recommend NHT [3, 4].

To the best of our knowledge, no studies have evaluated the pure effect of NHT itself on RM positivity and disease-free survival in the aspect of prostate-specific antigen (PSA) outcome, called biochemical recurrence (BCR), after all of the preoperative and intraoperative variables were conditionally corrected using matching. Therefore, this study aimed to evaluate the effect of NHT on RM positivity, BCR, and OS in patients with locally advanced PC who underwent RP. Patients were allocated into groups based on whether they were or were not administered NHT, and we used the propensity-score (PS) matching method to adjust for the different baseline clinicopathological variables between the two groups.

## 2. Materials and Methods

### 2.1. Ethical Statements

All study protocols were conducted according to the ethical guidelines of the “World Medical Association Declaration of Helsinki-Ethical Principles for Medical Research Involving Human Subjects.” This study was approved by the Institutional Review Board (IRB) of the Research Institute and Hospital National Cancer Center (IRB No. NCCNCS 05-049). All enrolled patients' informed consents were waived by the IRB.

### 2.2. Patients and Tissue Samples

From March 2004 to December 2015, 176 consecutive locally advanced patients with PC who underwent RP and were clinically staged at either ≥T3 or N+ at the Prostate Cancer Center within the National Cancer Center in Goyang, Korea, were retrospectively identified. Patients had complete medical records that included clinicopathological and prognostic information, such as follow-up duration of >6 months and survival outcomes. Those who did not reach a postoperative undetectable PSA level of <0.2 ng/mL were not included, as well as those who had a history of being treated with NHT for at least 3 months. Patients were then divided into NHT and non-NHT groups. Locally advanced PC was defined as having one or more of the following parameters: stage ≥T3 and/or PSA > 20 ng/mL and/or Gleason score sum 8-10; any stage T with pelvic nodal involvement; and clinical stage T3b or T4 disease without evidence of nodal involvement or metastasis [5, 6]. All final prostatectomy specimens were also reviewed according to the guidelines of the 2005 International Society of Urological Pathology (ISUP) by a single uropathologist (Dr. WSP) with 15 years of experience [7].

### 2.3. Statistical Analysis

The clinicopathological differences between NHT and non-NHT groups were evaluated using Pearson's *χ*2 test. The effect of NHT was evaluated by adjusting for significant clinicopathological variables, such as tumor volume percentage and extracapsular extension (ECE). One-to-one nearest neighbor matching using the PS matching technique was conducted with initial PSA level (<40 or ≥40 ng/dL), biopsied tumor volume percentage (<50 or ≥50%), age (<65 or ≥65 y), and Gleason score sum (<7 or ≥7) as matching variables. The effect of NHT was then re-evaluated in 50 matched pairs within the NHT and non-NHT group.

To investigate the effect of NHT on RM positivity, a univariable logistic regression analysis was performed before PS matching to identify variables with a significant association. Then, a multivariable logistic regression model was conducted using backward variable selection with an elimination criterion of* p*>0.05 applied. NHT and other clinical variables with* p*-value<0.2 in univariable analysis were entered the multivariable model. Last, a univariable logistic regression was performed after PS matching. To analyze survival, the Cox proportional hazards model was performed to examine the effect of NHT on BCR-free survival and OS. In this study, BCR was defined as a postoperative serum PSA elevation of >0.2 ng/mL assessed on two different occasions following a decrease to nondetectable level [8]. The first PSA value of ≥0.2 ng/mL was used to define the time of recurrence. The Cox proportional hazards models were also performed before and after PS matching.

The results were considered statistically significant when two-sided* p* values were <0.050. All analyses were performed using R project version 3.3.3 (The R Foundation, Vienna, Austria) and SAS version 9.4 (SAS Institute Inc., Cary, NC, USA).

## 3. Results

### 3.1. Patient Demographics

The overall median follow-up period, median BCR-free survival duration, and OS were 49.1 (range, 7.1-148.3) months, 42 (range, 1-48) months, and were not yet achieved, respectively. In the NHT group, the median treatment duration of NHT was 4 months.  Table 1 shows the distribution of clinicopathological variables in the NHT and non-NHT groups before and after PS matching. The results showed no differences in the distribution of preoperative clinicopathological factors between the two groups before PS matching, except for initial PSA level, ECE, lymphovascular invasion (LVI), perineural invasion, neurovascular bundle saving, and positive RM. After PS matching, only ECE, LVI, and neurovascular bundle saving were significantly different between the two groups. Other baseline patient demographics by group before and after the PS matching are summarized in Table 1.

### 3.2. Prognostic Factors for Resection Margin Positivity

The results from the univariable logistic regression model showed that before PS matching, NHT was a significant factor for predicting RM positivity (Table 2); however, it was no longer associated with RM positivity in the multivariate model. Conversely, tumor volume ≥50% and ECE were significant factors for RM positivity in both the univariable and multivariable models. After PS matching, NHT was no longer a significant factor for RM positivity.

### 3.3. Prognostic Factors for Biochemical Recurrence and Overall Survival

Before PS matching, NHT was not a significant prognostic factor for BCR in both univariable and multivariable Cox proportional hazards model results (Table 3). The initial PSA level, ECE, and LVI remained significant independent factors for BCR in the multivariable model. Even after matching, NHT was not a significant factor for BCR (*p*=0.554). As for OS, there were no significantly associated clinical variables before or after PS matching. Surprisingly, NHT failed to show a significant association with OS (Table 4).

## 4. Discussion

Discussions regarding the efficacy of NHT on RM and survival prognoses are typically met with the issue of different and heterogeneous baseline characteristics of enrolled patients with different tumor burdens in PC [9]. Our research group published two retrospective studies about the efficacy of NHT in PC [2, 10], showing that 75.4% of patients had pathologic RM negative, 19.5% had undergraded Gleason score, 50.0-57.1% of BCR rate, and 3.0-5.6% had nonrecurrent statuses with pT0 until a median follow-up of 59 months. However, both of these studies had a limitation of heterogeneous baseline characteristics.

In order to adjust for this imbalance in the baseline clinicopathological variables between patients with and without NHT, this study evaluated the effect of NHT on three main comparative endpoints (RM positivity, BCR, and OS) between NHT and non-NHT groups using PS matching in patients with locally advanced PC who underwent RP. Utilization of PS matching corrected the baseline differences for disease state and tumor burdens within the two groups and revealed the inefficacy of NHT on decreasing RM positivity, BCR, and OS, which was contrary to previous reports [2, 10, 11], and which was accorded to the current EAU guideline of PC [3]. The current EAU guideline recommended NHT only for patients with intermediate or high risk PC if receiving definitive radiation therapy.

The rationale for using NHT in PC was to decrease the size of the prostate volume by inhibiting the growth of hormone sensitive prostate cells and cancer cells. Decreasing prostate size might help clinicians to resect the prostate more efficiently and easily, with less intraoperative comorbidities during RP. However, the results of this study were consistent with those of previous studies that showed that HT did not completely eliminate the PC cells. Additionally, the disease states and the survival prognoses (BCR-free survival and OS) were not affected by NHT [12, 13]. Once androgen deprivation stops after discontinuing HT during intermittent HT, PSA level increases and the tumor may eventually regrow in the resection site. Other studies observed this process in patients with clear RM and without HT, who experienced recurrences at the operative site [1, 14]. However, some studies, including ours, have reported that patients achieved complete pathological response (about 0.2-5.4%) after HT, reflecting potential eradication of advanced prostate cancer with androgen deprivation therapy [2, 15, 16]. Patients with pT0 stage PC are expected to have an extremely favorable prognosis, especially in high risk or locally advanced PC. Further studies should be conducted in patients with pT0 after HT.

Previous systemic reviews and meta-analysis reports showed comprehensive assessments of the efficacy of HT when used as NHT with RP for treatment of PC [1]. Similar to this study, there was no improvement in clinically important outcomes for OS, disease-specific survival, or BCR-free survival when NHT was used with RP. This finding was observed despite improvements in putative pathologic surrogate outcomes, such as RM-free positive status (overall incidence in this study, 74.4%) and pathological downstaging (16.1%), particularly when NHT was maintained for >3 months (data not shown) [17].

In this study, the efficacy of NHT was not statistically significant; however, other studies have shown significant results for prognosis and RM, as well as some specific indicators for those patients with large tumor burdens [18–21]. In these circumstances, NHT should be cautiously discussed with patients and caregivers to make optimal treatment decisions, particularly because there are no phase III randomized controlled trials for NHT. For example, the use of NHT only prior to RP should be discussed in those patients with PC who have a larger prostate adenocarcinoma and a highly expected intraoperative and postoperative morbidity. A recent prospective 10-year NHT phase II study comparing large bulky PC (defined as a tumor >4 cm in diameter) or tumor involvement >50% of the gland with cancer and nonbulky PC evaluated this issue [21]. The study included 62 patients with T3 or T4N0M0 who were administered NHT with goserelin acetate and flutamide followed by RP. The patients achieved longer progression-free survival (7.5 years) and unreached OS (68% of 10-year OS) during a median follow-up time of 10.6 years. This result suggested that NHT might have a role in high volume locally advanced PC as an alternative to combined radiation and HT. In contrast, the present study had 90% of patients staged >T3, and PS matching showed no significant efficacy of NHT (Tables 2–4). The different results and interpretations between the two studies might be influenced by the androgen blocking agents, durations of NHT, and the adjuvant multidisciplinary and multimodal therapeutic options, such as long-term HT and external beam radiation therapy used after RP, which were not matched between the NHT and non-NHT groups in this study [22–24].

Additionally, administration of RP with NHT for 3 months might not be sufficient for improvement in disease-free survival compared with RP alone. Meyer et al. demonstrated that patients receiving NHT for more than 3 months had a significantly lower risk of PSA failure than the RP alone group [19]. In the work from the Canadian Urologic Oncology Group in 500 patients [25], a significant difference in BCR rates was not observed at 4 years, whereas continued pathologic benefit and BCR of PC were observed when NHT was used between 3 and 8 months. In the present study, we were unable to control the duration of NHT usage due to the retrospective design.

Another important factor affecting prognoses is the type of drugs used for NHT. In a recent phase II, randomized, open-label study of NHT with degarelix (gonadotropin-releasing hormone [GnRH] receptor antagonist) versus luteinizing hormone-releasing hormone (LHRH) agonist in patients with PC prior to RP, neoadjuvant degarelix alone was associated with higher levels of intratumoral dihydrotestosterone compared to the use of LHRH agonist and bicalutamide, despite similar testosterone levels [26]. Another randomized phase II trial in patients with intermediate and high risk PC who were administered NHT using LHRH agonists alone versus LHRH agonists plus abiraterone acetate prior to RP found that intraprostatic androgen levels of the prostate specimens were significantly reduced in the abiraterone plus LHRH agonist group than in the LHRH agonist alone group. Additionally, a 7.1% of pT0 rate and a 55.4% of residual cancer burden/minimal residual disease were reported for the entire cohort [27]. Heterogeneity of NHT drugs might be a confounding factor in the present study, and as such, future randomized controlled trials are required to clarify this issue.

Last important consideration for the use of HT is HT-associated complications. HT has its own local and systemic side effects, secondary to the blockade of androgen hormone [28, 29]. The side effects from HT are related to the duration of HT use. Even though short-term NHT before RP is typically used, the beneficiary effect of HT has been shown to occur after more than 3 months [1, 4, 18, 19, 25]. Clinicians should remain aware of the potential long-term side effects of HT, as severe liver function abnormality (17%), bowel problems (8%), and urinary and sexual (36%) problems have led to discontinuation of HT [30]. Therefore, further prospective, large-scale studies are needed to determine the true efficacy of NHT; however, the findings from this study do not support the use of NHT in locally advanced PC who underwent RP.

This study had limitations. First, this study had a small sample from a single institution with a short follow-up period. Second, the variations of the specific hormonal treatments, the duration of NHT, timing of treatments, the different antiandrogen agents and no adjustment of lymph node status or other pathological parameters in PS matching might have affected the results from the analyses. However, the clinical significance of this study is the potential to minimize the different baseline clinicopathological characteristics of tumor burden and disease status between groups using PS matching. Further studies with a large number of subjects and long-term follow-up are required, and the effect of secondary hormonal agents should also be investigated for their prognostic efficacy with and without androgen deprivation therapy.

## 5. Conclusion

This study showed that NHT was not a significant factor in predicting RM positivity, BCR-free survival, and OS before and after PS matching in patients with locally advanced or high risk localized PC who underwent RP.

## Figures and Tables

**Table 1:** Baseline characteristics before and after propensity score matching.

Characteristic	Before propensity score matching			After propensity score matching		
	RRP	RRP+NHT	*p* value	RRP	RRP+NHT	*p* value
	N(%)	N(%)		N(%)	N(%)	
No. of patients	97 (55.1)	79 (44.9)		50 (50.0)	50 (50.0)	
Age			0.710			0.410
<65	33 (34.0)	29 (36.7)		17 (34.0)	21 (42.0)	
≥65	64 (66.0)	50 (63.3)		33 (66.0)	29 (58.0)	
PSA			**<.001**			1.000
<40	84 (86.6)	37 (46.8)		37 (74.0)	37 (74.0)	
≥40	13 (13.4)	42 (53.2)		13 (26.0)	13 (26.0)	
Biopsy Gleason score sum			0.476			0.401
<7	16 (16.5)	10 (12.7)		9 (18.0)	6 (12.0)	
≥7	81 (83.5)	69 (87.3)		41 (82.0)	44 (88.0)	
Tumor volume			0.178			1.000
<50%	62 (63.9)	58 (73.4)		31 (62.0)	31 (62.0)	
≥50%	35 (36.1)	21 (26.6)		19 (38.0)	19 (38.0)	
Clinical T stage						
2	1 (1.0)	3 (3.8)		1 (2.0)	1 (2.0)	
≥3	96 (99.0)	76 (96.2)		49 (98.0)	49 (98.0)	
Clinical N stage						
0	90 (92.8)	57 (72.1)		45 (90.0)	34 (68.0)	
1	7 (7.2)	22 (27.9)		5 (10.0)	16 (32.0)	
Extracapsular extension	60 (61.9)	30 (38.0)	**0.002**	34 (68.0)	22 (44.0)	**0.016**
Seminal vesicle invasion	37 (38.1)	33 (41.8)	0.625	21 (42.0)	20 (40.0)	0.839
Lymphovascular invasion or emboli	32 (33.0)	9 (11.4)	**0.001**	17 (34.0)	6 (12.0)	**0.009**
Perineural invasion	79 (81.4)	52 (65.8)	**0.018**	40 (80.0)	37 (74.0)	0.476
Apex involvement	25 (25.8)	11 (13.9)	0.053	13 (26.0)	7 (14.0)	0.134
Lymph node dissection	86 (88.7)	76 (96.2)	0.066	43 (86.0)	49 (98.0)	0.059
Neurovascular bundle saving	47 (48.5)	17 (21.5)	**<.001**	25 (50.0)	12 (24.0)	**0.007**
Resection margin	36 (37.1)	16 (20.3)	**0.015**	19 (38.0)	11 (22.0)	0.081
Death	7 (7.2)	11 (13.9)	0.144	6 (12.0)	7 (14.0)	0.766

The *p* value was calculated using Pearson's chi-squared test between NHT and non-NHT groups.

**Table 2:** Logistic regression model for resection margin positivity before and after propensity score matching.

Variables	Before propensity score matching				After propensity score matching	
	Univariable analysis		Multivariable analysis		Univariable analysis	
	OR (95% CI)	*p* value	OR (95% CI)	*p* value	OR (95% CI)	*p* value
NHT plus RRP versus RRP alone	**0.43 (0.22-0.86)**	**0.016**	**0.59 (0.27-1.30)**	**0.188**	**0.46 (0.19-1.11)**	**0.084**
Age ≥65	0.82 (0.42-1.60)	0.561			0.73 (0.30-1.74)	0.473
PSA ≥40	1.41 (0.71-2.79)	0.328			4.23 (1.64-10.93)	0.003
Biopsy Gleason score sum ≥7	1.47 (0.56-3.91)	0.436			1.86 (0.49-7.15)	0.365
Tumor volume ≥50%	6.67 (3.26-13.62)	<.001	3.68 (1.67-8.09)	0.001	5.78 (2.28-14.63)	<.001
Extracapsular extension	9.33 (4.04-21.53)	<.001	5.26 (2.13-12.96)	<.001	6.29 (2.16-18.33)	0.001
Seminal vesicle invasion	2.86 (1.47-5.58)	0.002			3.07 (1.27-7.42)	0.013
Lymphovascular invasion or emboli	4.67 (2.23-9.80)	<.001			4.59 (1.71-12.28)	0.002
Perineural invasion	5.93 (2.00-17.57)	0.001			2.42 (0.75-7.86)	0.141
Apex involvement	253.23 (32.55-.)	<.001			NA	
Lymph node dissection	1.05 (0.32-3.52)	0.934			0.69 (0.15-3.10)	0.631
Neurovascular bundle saving	0.55 (0.27-1.11)	0.094			0.51 (0.20-1.31)	0.165

**Table 3:** Cox proportional hazards model for biochemical recurrence before and after propensity score matching.

Variables	Before propensity score matching				After propensity score matching	
	Univariable analysis		Multivariable analysis		Univariable analysis	
	HR (95% CI)	*p* value	HR (95% CI)	*p* value	HR (95% CI)	*p* value
NHT plus RRP versus RRP alone	**1.05 (0.69-1.61)**	**0.809 **	**1.03 (0.62-1.72)**	**0.906 **	**0.84 (0.47-1.49)**	**0.554 **
Resection margin	1.51 (0.97-2.36)	0.071			2.14 (1.18-3.86)	0.012
Age ≥65	0.72 (0.46-1.10)	0.128			0.61 (0.34-1.08)	0.091
PSA≥40	2.14 (1.40-3.29)	0.001	2.23 (1.38-3.60)	0.001	3.19 (1.77-5.76)	<.001
Biopsy Gleason score sum ≥7	2.35 (1.13-4.87)	0.022			2.77 (0.99-7.73)	0.052
Tumor volume ≥50%	1.78 (1.15-2.75)	0.010			2.50 (1.41-4.44)	0.002
Extracapsular extension	2.47 (1.59-3.85)	<.001	2.10 (1.30-3.41)	0.003	5.04 (2.48-10.23)	<.001
Seminal vesicle invasion	2.20 (1.44-3.36)	<.001			2.42 (1.36-4.30)	0.003
Lymphovascular invasion or emboli	2.35 (1.50-3.68)	<.001	1.85 (1.13-3.05)	0.015	3.32 (1.83-6.00)	<.001
Perineural invasion	2.04 (1.18-3.51)	0.011			3.61 (1.43-9.17)	0.007
Apex involvement	1.71 (1.05-2.78)	0.031			2.39 (1.26-4.54)	0.008
Lymph node dissection	1.09 (0.47-2.49)	0.845			2.28 (0.55-9.41)	0.256
Neurovascular bundle saving	0.62 (0.39-0.99)	0.045			0.56 (0.30-1.05)	0.070

**Table 4:** Cox proportional hazards model for overall survival before and after propensity score matching.

Variables	Before propensity score matching		After propensity score matching	
	Univariable analysis		Univariable analysis	
	HR (95% CI)	*p* value	HR (95% CI)	*p* value
NHT plus RRP versus RRP alone	**1.35 (0.52-3.51)**	**0.539**	**0.77 (0.25-2.38)**	**0.650**
Resection margin	1.62 (0.60-4.38)	0.338	1.94 (0.63-5.98)	0.250
Age ≥65	1.27 (0.45-3.55)	0.654	1.29 (0.40-4.22)	0.669
PSA≥40	1.04 (0.39-2.77)	0.943	0.67 (0.15-3.04)	0.599
Biopsy Gleason score sum ≥7	1.36 (0.39-4.75)	0.630	0.84 (0.23-3.10)	0.795
Tumor volume ≥50%	2.17 (0.82-5.77)	0.120	3.01 (0.97-9.28)	0.056
Extracapsular extension	1.03 (0.41-2.62)	0.948	1.22 (0.41-3.65)	0.717
Seminal vesicle invasion	1.84 (0.71-4.77)	0.210	1.53 (0.48-4.86)	0.471
Lymphovascular invasion or emboli	0.45 (0.10-1.95)	0.286	0.70 (0.16-3.17)	0.644
Perineural invasion	2.69 (0.77-9.34)	0.119	2.22 (0.49-10.09)	0.301
Apex involvement	1.59 (0.51-4.94)	0.421	3.02 (0.87-10.53)	0.083
Lymph node dissection	NA		NA	
Neurovascular bundle saving	0.62 (0.22-1.75)	0.370	0.69 (0.21-2.23)	0.532

## Data Availability

The datasets used and/or analysed during the current study available from the corresponding author (Kang Hyun Lee, uroonco@ncc.re.kr) on reasonable request. The IRB and ethical committee of the National Cancer Center (in Korea) will review the requests because of the patients' information. After the approval of the committee with confirmation of the reasonable requests, the dataset will be freely available. The other contact e-mail besides the corresponding author's e-mail is irb@ncc.re.kr.
